# Barriers of effective health insurance coverage for rural-to-urban migrant workers in China: a systematic review and policy gap analysis

**DOI:** 10.1186/s12889-020-8448-8

**Published:** 2020-03-30

**Authors:** Shanquan Chen, Yingyao Chen, Zhanchun Feng, Xi Chen, Zheng Wang, Jianfeng Zhu, Jun Jin, Qiang Yao, Li Xiang, Lan Yao, Ju Sun, Lu Zhao, Hong Fung, Eliza Lai-yi Wong, Dong Dong

**Affiliations:** 1 The School of Clinical Medicine, University of Cambridge, Cambridge, UK; 2 School of Public Health, Fudan University, Shanghai, China; 3 School of Medicine and Health Management, Tongji Medical College, Huazhong University of Science and Technology, Wuhan, China; 4 Department of Health Policy and Management, Yale School of Public Health, New Haven, Connecticut USA; 5 Research Center for Healthcare Management, School of Economic and Management, Tsinghua University, Beijing, China; 6 Guizhou Provincial Institute of Health Development, Guizhou Medical University, Guiyang, Guizhou China; 7 School of Social Development and Public Policy, Fudan University, Shanghai, China; 8 Department of Sociology, Tsinghua University, Beijing, China; 9 School of Political Science and Public Administration, Wuhan University, Wuhan, Hubei China; 10 Health Bureau, Macao SAR, China; 11 State Key Laboratory for Diagnosis and Treatment of Infectious Diseases, National Clinical Research Center for Infectious Diseases, Collaborative Innovation Center for Diagnosis and Treatment of Infectious Diseases, The First Affiliated Hospital, College of Medicine, Zhejiang University, Hangzhou, Zhejiang China; 12 The Jockey Club School of Public Health and Primary Care, Faculty of Medicine, The Chinese University of Hong Kong, Hong Kong SAR, China

**Keywords:** China, Rural-to-urban migrant workers, Universal health coverage, Systematic review

## Abstract

**Background:**

More than 90% of the Chinese population was covered by its three basic social health insurances. However, the Chinese rural-to-urban migrant workers (RUMWs), accounting for about one-fifth of China’s total population, seem to be put on a disadvantaged position under the current health insurance schemes. The purpose of this study is to identify the current barriers and to provide policy suggestions to the ineffective health insurance coverage of RUMWs in China.

**Methods:**

A systematic review guided by the Preferred Reporting Items for Systematic Reviews and Meta-Analysis (PRISMA) guidelines. The searched databases included PubMed, Embase, Medline, Web of Science, PsycINFO, Maternity and Infant Care Database MIDIRS, the Cochrane Library, WHO Library Database (WHOLIS), WHO Global Health Library, World Bank eLibrary, OpenGrey, CNKI, and Wanfang. In total, 70 articles were reviewed.

**Results:**

(1) Chinese RUMWs have high work mobility and low job stability; (2) Barriers faced by RUMWs in obtaining effective health insurance coverage are primarily due to the reluctance of employers to provide insurance for all employees and the disadvantaged position held by RUMWs when negotiating with their employers; (3) Fissures among existing health insurance schemes leaves no room for RUMWs to meet their primary needs; and (4) Recent efforts in improving the portability and transferability of insurance across borders and schemes are not enough to solve the barriers.

**Conclusion:**

It is argued that the Chinese central government must deal with the fragmentation of healthcare system in China and promote effective coverage by: (1) playing a more active role in coordinating different healthcare and social welfare schemes across the country, (2) increasing the health insurance portability, (3) making the healthcare policies more compatible with RUMW’s characteristics to meet their primary health needs, (4) strengthening supervision of employers, and (5) providing more vocational training and other support to increase RUMW’s job stability.

## Background

Universal health coverage (UHC) is a vision where all people and communities have access to quality healthcare services where and when they need them, without suffering financial hardship [1, 2]. The concept of UHC is in line with China’s health reform launched in 2009, which announced a provision of affordable and equitable basic health care for all by 2020 [3]. In October 2016, another ambitious plan—healthy China 2030—advances the concept of UHC in China from pursuing widespread coverage to effective coverage [4].

As early as 2010, more than 90% of the Chinese population was covered by its three basic social health insurances (SHI) [5], namely New-rural Cooperative Medical Scheme (NCMS) for rural residents, Urban Resident-based Basic Medical Insurance (URBMI) for urban residents, and Urban Employee-based Basic Medical Insurance (UEBMI) for formal urban workers) [6]. Their role in increasing health service accessibility, reducing economic burden, and improving health equity are evidential [5, 7].

However, the studies during 2014 and 2018 indicated that the Chinese rural-to-urban migrant workers (RUMWs)—villagers who migrate to urban areas for employment opportunities—seem to be put on a disadvantaged position in UHC. Their effective health insurance coverage is low [8–13], largely because they are geographically removed from their place of insurance registration. Meanwhile the insurance provided by their workplace is often insufficient or even absent. Consequently, it is not uncommon for RUMWs to use no treatment [14–20], self-treatment [15–22] or informal health services [14–16, 19, 23–25] when they are sick. The problem of geographical disjunction from the health insurance register and usage makes health services less accessible for RUMWs [14, 16, 19–21, 23, 26–32] or they have to face higher health economic burden compared with urban or even rural residents [9, 11, 14–16, 21, 23, 33–35].

Eliminating this geographical disjunction in access to health insurance has been the primary goal of the creation and revision of health policies related to RUMWs by two Chinese government policies, issued in 2010 [36] and 2016 [37] respectively. However, studies have shown that, even though RUMWs live in urban areas, they are still greatly marginalized by the urban health system [8, 12, 26, 38–40]. The percentage of RUMWs covered by health insurance in their flow-in areas has fluctuated between 18 and 20% since 2008 [41]. This low effective coverage and fluctuating percentage is frequently attributed to the defect in the design of China’s health insurance system.

RUMWS usually take jobs shunned by urbanites and contribute significantly to urban development [14, 38]. By 2017, there were 286.5 million RUMWs, accounting for about one-fifth of China’s total population [41]. The huge population of RUMWs directly influences the achievement of UHC or the effectiveness of UHC in China. Therefore, to facilitate better implementation of the UHC on RUMWs, a systematic review was conducted. Primarily focusing on the effectiveness of health insurance coverage, our review is composed of five parts: 1) the characteristics of RUMWs and the features of their health needs, 2) the barriers faced by RUMWs in obtaining effective health insurance coverage, 3) the policy gaps in existing efforts to solve the barriers, and 4) domestic and international innovative approaches that can be helpful in improving the effective health insurance coverage for RUMWs. In the final section, we propose potential strategies on how to overcome the current barriers and make changes to the ineffective health insurance coverage of RUMWs in China.

## Methods^1^

The aim of the study is three-fold: (1) to review China’s healthcare policies and their applications to rural-to-urban migrant workers (RUMWs) in China; (2) to identify problems faced by RUMWs and the policy gaps that need to be addressed in future; and (3) to facilitate better implementation of the UHC on RUMWs.

### Search strategy

For the systematic review, we used the steps recommended in the Preferred Reporting Items for Systematic Reviews and Meta-Analysis (PRISMA) guidelines. We searched PubMed, Embase, Medline, Web of Science, PsycINFO, Maternity and Infant Care Database MIDIRS, the Cochrane Library, WHO Library Database (WHOLIS), WHO Global Health Library, World Bank eLibrary, OpenGrey, CNKI (zhiwang, 知网, a major Chinese academic publication consolidation database), and Wanfang (万方, another major Chinese academic database) for published or unpublished papers and reports in English or Chinese between Jan 1, 2008, and Dec 31, 2018. We searched for studies published after Jan 1, 2008, because the situation in China has been changing rapidly, and the information provided by older studies may already be detached from their original research contexts and be less useful for further work. The research strategy was developed based on similar reviews in other settings [42–45]. The search terms used controlled vocabulary and free text, which included word combinations intended to capture a variety of Chinese and English texts depicting Nongmingong (农民工, in Chinese, literally meaning peasant worker) and rural-to-urban migrants (in English, e.g., Migra* or Transient* or Emigra* Peasant* or Newcom* or New-com* or “Mobil* population” or “Mobil* people” or “Mobil* work*” or “Float* population*” or “Float* people” or “Float* work*).

### Inclusion and exclusion criteria

The inclusion and exclusion criteria are primary twofold. First, we included studies that offer the information related to accessibility, acceptability, affordability, and availability in the view of RUMWs [46–48] and “six building blocks” in the view of health system [49] – service delivery, health workforce, health information systems, access to essential medicines, financing and leadership/governance. Second, only original research was included; comments, correspondence, and editorials were excluded.

### Quality assessment

Quality assessment was conducted as follows: The quality of the observational cohort/cross-sectional studies and case-control studies was assessed using an adaptation of Study Quality Assessment Tools (SQAT) developed by the US National Institutes of Health (NIH) [50]. The quality of the qualitative studies was assessed using an adaptation of the Critical Appraisal Skills Programme (CASP) quality-assessment tool [51]. Each of the quantitative findings was assessed using the GRADE (Grading of Recommendations Assessment, Development, and Evaluation) approach [52], and each of the qualitative findings was assessed using the GRADE-CERQual (Confidence in the Evidence from Reviews of Qualitative Research) approach [53].

The detailed process can be found in supplement s1.

## Results

**Fig. 1:**
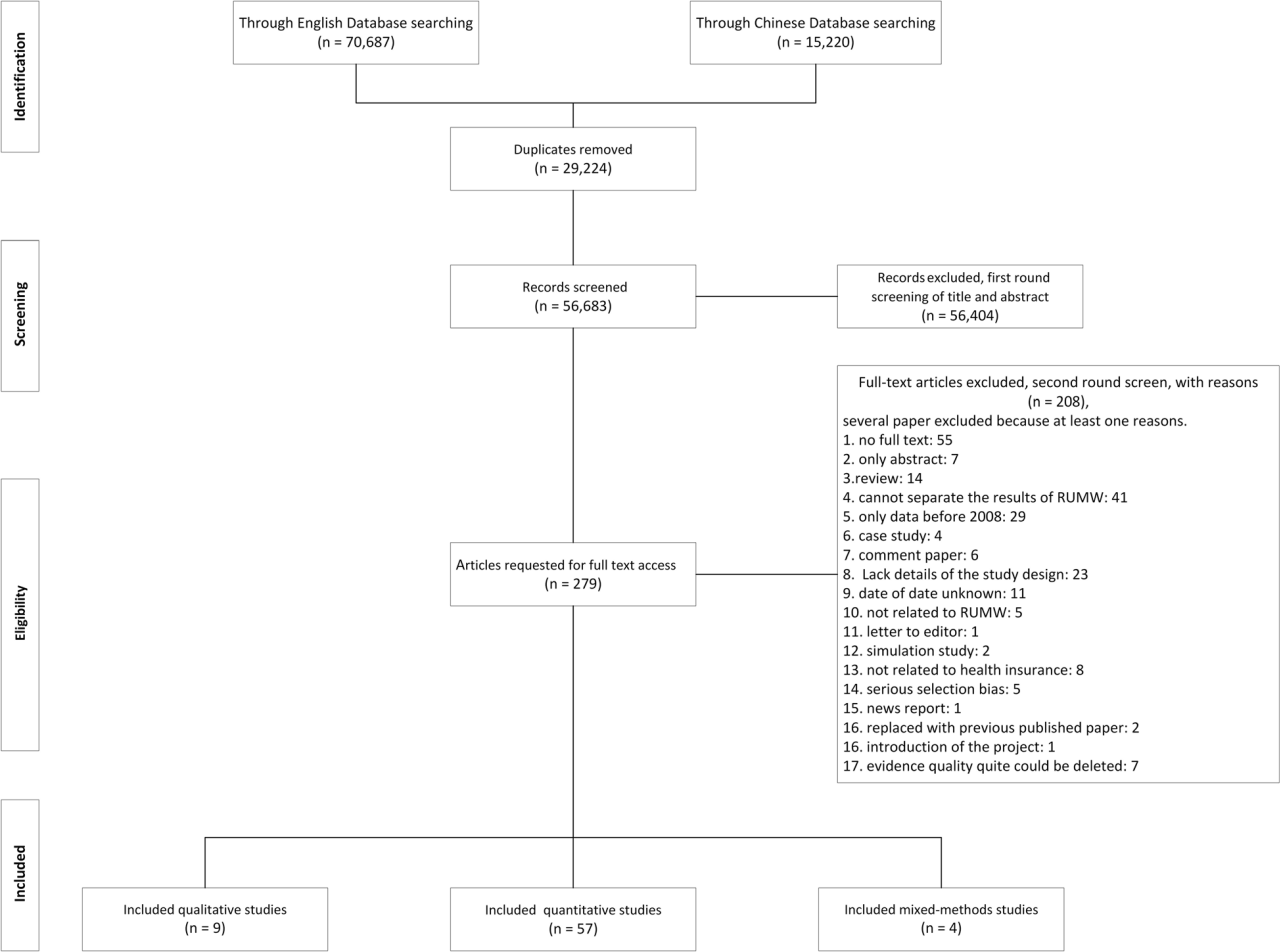
Flowchart of the selection of studies included in the review of barriers of effective health insurance coverage for rural-to-urban migrant workers in China

Through our searches, 70,687 records were identified from the English database, and 15,220 records were identified from the Chinese database. After removing 29,224 duplicated records, 56,683 records were screened, of which 279 full-text articles were assessed for eligibility, and 70 articles that fit the standards were included in this study (Fig. 1).

### Characteristics of RUMWs and features of their health needs

RUMWS have long been considered a vulnerable group due to their poor education [14, 21, 26, 40], poor living conditions [14, 15], long working hours [14, 21, 40], and low income [14, 40]. They lack social integration in the city [14, 15, 34, 54], and mainly rely on their kinship and friendships for social support [14, 34]. More than half of them move across provinces (27.2%) or across cities (32.9%) when they are young [55], but inevitably return to their hometown when they are too aged or ill to support their floating life [40]. They usually do not have special skills, and typically take temporary work in private sectors because state sectors usually reserve jobs for locals or skilled workers [14, 19, 40, 56, 57]. A majority of RUMWs are discriminated and treated as a low-cost labor force. But sometimes they are also acclaimed as contributors to urbanization and economic development [14, 16, 17, 28, 33, 38, 58]. Therefore, most RUMWs have high work mobility and low job stability, placing them in a disadvantaged and marginalized socioeconomic position [33].

The inherent characteristics of RUMWs inevitably shape their health needs, as follows: (1) They exhibit better physical health [17, 19, 40, 59, 60], but worse mental health than local residents [17, 19, 34, 59, 2) Except for industrial injury, they are less likely to suffer from serious diseases, but are more likely affected by common ailments, infectious diseases [15, 60–62] or sexually transmitted diseases [3, 63] Their needs for healthcare and medical services are often delayed and the origins of their illness are often far from where they end up [14, 34], as they devote their young and healthy bodies to the flow-in cities, and bring their older and ailing bodies back to the flow-out place [40]. The question is: What factors contribute to the suffering of RUMWs in China within the current healthcare system? Through a review of existing literature, we have identified two major barriers.

### Barriers to effective health insurance coverage among RUMWs

#### Barrier 1: difficulties of RUMWS being included in the healthcare system in the flow-in areas

Due to historical reasons, the Chinese welfare system is mainly financed at the local level [38, 64]. However, for local governments, even today, the local GDP growth rate is one of the most important political performance indicators [38]. Therefore, local governments are caught in a dilemma. On one hand, they need to expand welfare coverage to attract skilled migrants to contribute to local economic growth, but this raises the labor costs and increases local finance burdens [38]. On the other hand, they are reluctant to bear excessive financial burden because they need to control labor costs to attract investment funds [38]. To compromise effectively, the local government expanded the welfare coverage to the RUMWs with the desired skills and qualifications [33, 38, 64].

Business sectors face a dilemma similar to the local governments [65]. Since both the employee and the employer need to contribute to the employee’s welfare account, the high work mobility and low job stability of RUMWs could increase the burden for the employers [38]. Further, the Labor Contract Law bundles the health insurance premium with other welfare programs, such as pension insurance, unemployment insurance, on-the-job injury insurance, maternity insurance, and housing provident fund [38, 58, 65–67]. This bundling leads to an increased burden for local governments and business sectors [38, 65]. As a result, the business sectors—especially private enterprises [64, 67] and small and medium-sized enterprises (SMEs) [68]—have adopted a similar strategy trying to avoid providing insurance coverage to all RUMWs [64, 65, 69, 70].

The local governments have now developed greater financial capacities to accommodate people that they had excluded before, but due to a lack assistance from government or nongovernment organizations [15, 28, 57] coupled with their disadvantaged and marginalized socioeconomic status, RUMWs remain in a weak position during negotiations with their employers [8, 64, 69]. Even when RUMWs sign a formal contract with their employers, they are sometimes hired by subcontractors or labor dispatch companies, and thus their labor relationship with their true employers are not clear and cannot be fully protected by the Labour Contract Law [14, 15, 57, 67]. Similarly, in cases where the employers refuse to pay premium for RUMWs [26, 65], the employer’s accountability is not supervised or well-regulated [15, 26, 67].

#### Barrier 2: fissures among existing health insurance schemes leaves no room for RUMWs to meet their primary needs

The evidence generated during 2013 and 2018 indicated that due to the fragmentation of its healthcare system [15, 38, 69, 70], health insurance portability or transfer in China is low [38, 64, 71–74]. More recently, there have been reforms in both the healthcare and social security sectors, which lead toward the integration of NCMS and URBMI [75, 76]. Through this, the fragmentation of China’s health system should be greatly reduced. Yet the fissures between NCMS and URBMI or UEBMI still exist. Specific barriers that hinder the portability of health insurance are elaborated below (summary in Table 1).

1. ***Low portability between URBMI and NCMS for RUMWs****.* “Health insurance portability” means that an insurance holder can transfer his/her insurance from one plan to another plan, and from one place to another place [73, 80]. As the World Bank report of “The Path to Integrated Insurance Systems in China” [81] suggests, the integration of NCMS and URBMI will increase the portability of both NCMS and URBMI. However, both insurances are registered based on the unit of family, while most RUMWs migrate to cities without their families [57, 65]. Participating in URBMI in the flow-in place will leave their families uninsured. Whereas the left-behind elderly family members are often the primary users of NCMS in the flow-out place [65]. This dilemma leaves the RUMWs no choice but to keep NCMS for their families and leave themselves uninsured.
2. ***Incompatibility between UEBMI and NCMS for RUMWs****.* Both UEBMI and NCMS have a risk-sharing account, and UEBMI also has an individual account funded by the employees such as the RUMWs. The risk-sharing account for UEBMI is funded by the employers, while the one for NCMS is funded by both the government and the family. Contributions from the employers and the local governments have a strong and direct influence on the affordability and sustainability of the local social welfare system. Therefore, by no means are the local governments willing to transfer out the funds in the risk-sharing accounts of UEBMI or the funds paid by the government in NCMS [71, 80]. Currently, only the funds in the individual account of UEBMI and the funds contributed by the family in NCMS [71, 80] are portable. Moreover, even if the RUMWs are allowed to transfer from NCMS to UEBMI, they are less likely to do so due to the economic burden induced by the high premiums for UEBMI [14, 38, 68, 69, 82].
3. ***Including RUMWs into the migrant work health insurance (MWHI)***. MWHI is a plan specifically designed to solve the health needs of the increasing number of migrant workers. Despite the variance of MWHI across regions, the evidence generated during 2009 and 2010 indicated that almost all MWHIs are featured by low premium, mandatory employer contribution, and inpatient first [14, 83]. MWHI considers the low income of RUMWs, but it is still a voluntary program and only effective after signing a formal labor contract. Therefore, as outlined in Barrier 1, issues such as reluctance of the employers to offer health insurance for RUMWs also applies to MWHI. Additionally, two studies from 2010 and 2013 indicated that only offering risk protection for inpatient services is essentially a mismatch with the health needs of RUMWs [70, 84]. Two studies from 2013 and 2015 indicated that MWHI has almost zero portability, which is also incompatible with the RUMWs’ high place mobility and low job stability [70, 77].
4. ***Keeping the RUMWs covered by the NCMS in their flow*-*out place.*** In fact, this is the option chosen by most RUMWs [19, 26, 85–87]. About 60% of RUMWs stay in the NCMS in their flow-out place, as shown in Table 1. However, the use of NCMS is largely bounded by geography. With the development of NCMS, it has become normal for NCMS to cover services beyond their municipal or provincial boundaries. However, most of the evidence updated to 2018 indicated that only hospital services are reimbursed through NCMS across borders and no primary health services are included [88, 89]. Thus, the fundamental health needs of RUMWs cannot be met by this mechanism.

**Table 1:** Comparison of health insurance currently available for rural-to-urban migrant workers

	NCMS	URBMI	UEBMI	Early MWHI
**Launch year**	2003	2007	1998	2006
**Eligible conditions**				
Eligible population	Rural, employed/non-employed	Urban, non-employed	Urban/rural, employed/self-employed	rural in urban, employed
contract	–	–	Necessary	Necessary
Dependence on employee	–	–	Yes	Yes
**Coverage rate**^a^				
In flow-out area	57.6% + 3.5%	3.4% + 3.5%	3.0%	< 0.7%
In flow-in area	6.7% + 1.4%	3.7% + 1.4%	18.6%	< 1.5%
Total	64.7% + 4.9%	7.2% + 4.9%	21.9%	< 2.2%
**Insurance type**^b^	Limited-duration health insurance	Limited-duration health insurance	Limited-payment whole-life health insurance	Limited-duration health insurance
Guarantee period	The following one year	The following one year	The following one year and future	The following one year
**Account type**	Risk-sharing account	Risk-sharing account	Individual account + risk-sharing account	Risk-sharing account
**Financing strategy** [6]				
Minimum financing unit	Family	Family	Employed: employee + employer Self-employed: individual	Employed: employee or + employer Self-employed: individual
Financing contribution rate	Family: 20% Government subsidy:80%	Family: 30% Government subsidy:70%	8% of payroll Employed: employee: 2% employer: 6% Self-employed: 8%	Low
Total financing amount per unit (RMB/year)	NA	NA	3485 * 12 * 8% = 3345.6	Low
Bundled with other welfare programs	NA	NA	Pension insurance On-the-job injury insurance Unemployment insurance Maternity insurance Urban Minimum Standard Living Allowance Program Housing provident fund	Yes or no
Employer’s total contribution rate [38]	–	–	Approximately 30%	Low
Employee or individual’s total contribution rate [38]	–	–	Employed: Approximately 10%Self-employed: 8%	Low
Total amount of burden for funders per unit (RMB/year/people) ^c^	Family: 220 Government subsidy: 490	Family: 220 Government subsidy: 490	Employed: employee: 3485 * 12 * 10% = 4182 employer: 3485 * 12 * 30% = 12,546 Self-employed: 3485 * 12 * 8% = 3345.6	Low
**Covered services**	flow-out area: Outpatient + Inpatient flow-in area: Inpatient	Outpatient + Inpatient	Outpatient + Inpatient	Inpatient
Matching with RUMWs’ health need	Mismatch	Match	Match	Mismatch
**Geographic consistency for RUMWs**	geographically separated	geographically consistent	geographically consistent	geographically consistent
**Portability, in the view of RUMWs** [64, 70, 73, 77]	Low	Low	Quite low	No
**Membership selectivity** [38, 64, 71]				
Employee	–	–	High	Low
Government	Low	Low	High	–

^a^The percentage is calculated from China Migrants Dynamic Survey, a national survey covering about 78 thousand RUMWs [78]. Several places integrated NCMS and URBMI in to one. The percentage after the plus sign is of those who took part in the integrated NCMS and URBMI

^b^ Limited-payment whole-life health insurance refers to the insurance plan that has a set period, in which an insurance holder pay premiums into the policy. Once the holder reaches the target years, premiums are no longer required but the policy’s benefits lasts the insured’s entire life. Limited-duration health insurance refers to a plan with a limited duration, paid by years or less

^c^ “220” is drawn from the payment standards for basic medical insurance of urban and rural residents in 2018 [79]; “3458” from the average payroll of RUMWs in 2017 [41]

### Policy gaps in existing solutions to increase effective health insurance coverage of RUMWs

Two new reforms are linked to the benefits of RUMW: the implementation of *Interim Measures for the Transfer and Continuation of Basic Medical Security Relationships of Migrant Employees* (launched in 2010 and modified in 2016, 36, 37] and the integration of WMHI into UEBMI. However, both solutions have problems that prevent the RUMWs being effectively covered by health insurance.

#### Policy gap 1: lacking detailed policies has exacerbated fragmentation and is not helpful for health insurance portability

The *Interim Measures* is a national level solution to the geographical exclusion caused by mobile employment, which allows insurance transfers between regions or between plans. It is an important policy for achieving effective coverage of the Chinese UHC. In the 2010 version of the *Interim Measures* [36], the stipulations related to RUMWs are: 1) no double coverage by the three basic SHI (i.e., NCMS, URBMI, and UEBMI); 2) the government flow-in area cannot refuse the migrant worker from taking part in the local SHI using the excuse of hukou^2^; 3) the RUMW, who has a stable labor relationship with a local institution, should be covered by local UEBMI; 4) the RUMW, who has an unstable labor relationship with a local institution, can voluntarily chose to keep their insurance in the flow-out place or to utilize the local basic health insurance; and 5) when the RUMWs return and if they still hold the rural hukou, they need to re-transfer their insurance back to the NCMS in their hometown.

However, none of these five stipulations consider the dilemmas faced by RUMWs illustrated above. The fifth stipulation can even cause loss in benefits to the RUMWs if they are covered by the UEBMI in their flow-in place. In addition, a lack of details in the *Interim Measures* has exacerbated the fragmentation among local policies since different regions have developed their own operational approaches for the SHI relationship transfer [71]. Studies also show that the transfer of health insurance for migrant workers does not work well [74, 80], especially for those who have high mobility [70, 90, 91]. Finally, what is noteworthy is that the ideas behind second and fifth stipulation are against with each other. The former one tries to weaken the influence of hukou, while the later one actually strengthens it.

In 2016, the *Interim Measures* were modified [37]. The newer version deletes the above five stipulations and seems to increase the mutual portability among three SHI. But how to understand and implement the *Interim Measures* almost completely depend on the local government. Due to the lack implementation details, local governments who have already formed their own rules are less like to revise or make the implementation more effectively [38]. More critically, the same as the 2010 version, the 2016 version does not touch the risk-sharing accounts that are highly related to the benefits of the local government. Problems elaborated in Barrier 2 are not tackled and the RUMWs’ primary health needs are not dealt with.

Another problem for current policies is the ambiguous description of eligibility. For most policies, the eligibility of local health insurance for the RUMWs is based on stable labor relations. However, what the stable means is not clear [38, 83]. This also gives the employer a chance to evade their responsibility, if they recruit workers from subcontractors or labor dispatch companies [14, 15, 57]. An additional question is which insurance self-employed RUMWs are eligible for, WMHI, UEBMI, or URBM? The related description about them are usually absent [14, 70, 83]. For instance, in Table 2, we will introduce next, none of Beijing, Shanghai and Shenzhen gave a clear statement.

**Table 2:** comparison of before and after the reform on MWHI in Beijing, shanghai and Shenzhen

	Shangha i[92–96]		Beijin g[97–99]		Shenzhe n[100–102]			
	Before 2011	After 2011	Before 2012	After 2012	Before 2014	After 2014		
						Category I	Category II	Category III
**Eligible conditions**								
Eligible population	Non-local workers	Local/non-local workers	Non-local workers	Local/non-local workers	Non-local workers	Local/non-local workers	Non-local workers	
Contract	Unnecessary	Necessary	Necessary	Necessary	Necessary	Necessary		
Dependence on employee	No	Yes	Yes	Yes	Yes	Yes		
**Insurance type**^a^	Limited-duration health insurance	Limited-payment whole-life health insurance	Limited-duration health insurance	Limited-payment whole-life health insurance	Limited-duration health insurance	Limited-payment whole-life health insurance		
Guarantee period	The following one year	The following one year and future	The following one year	The following one year and future	The following one year	The following one year and future		
**Account type**	Individual account + risk-sharing account	Individual account + risk-sharing account	Risk-sharing account	Individual account + risk-sharing account	Risk-sharing account	Individual account + risk-sharing account	Risk-sharing account	
**Management agency**	Commercial insurance company	Social insurance agency	Social insurance agency	Social insurance agency	Social insurance agency	Social insurance agency		
**Financing strategy**								
Minimum financing unit	Employed: employee Self-employed: individual	Employed: employee + employer	Employer	Employee + employer	Employee + employer	Employee + employer		
Financing contribute rate	12.5% (non-local construction enterprise rate is 5.5%)	Employee: 9.5% Employer: 2%	Employer: 2%	Employee 2% + 3 RMB Employer: 10%	Employee: 4 RMB/month Employer: 8 RMB/month	Employee 2% Employer: 5.2% or 6.2%	Employee 0.2% Employer: 0.6%	Employee 0.1% Employer: 0.45%
Total financing amount per unit (RMB/year)	3485 *12 * 12.5% or 5.5%	3485 *12* 11.5%	3485 *12 * 2%	3485 *12 * 12% + 36	12* 12	3485 *12 * 7.2% or 8.2%	3485 *12 * 0.8%	3485 *12 * 0.55%
Bundled with other welfare programs	Pension insurance On-the-job injury insurance	Same with UEBMI	NA	Same with UEBMI	NA	Same with UEBMI		
Employer’s total contribution rate	12.5% (non-local construction enterprise rate is 5.5%)	31.2–32.9%	2%	30.8–32.5%	8 * 12 RMB	18.49–20.49%	15.16–16.16%	14.74–15.74%
Employee or individual’s total contribution rate	None	10.50%	None	10.2% + 3RMB	4 * 12 RMB	10.3%	8.5%	8.4%
Total amount of burden by funders per unit (RMB/year)	Employed: employee: None employer: 2300 or 5228 Self-employed: 5228	Employed: employee: 4391 employer: 13,048–13,786 Self-employed: Unclear	Employed: employee: None employer: 836 Self-employed: Unclear	Employed: employee: 4320 employer: 12,881–13,592 Self-employed: Unclear	Employed: employee: 48 employer: 96 Self-employed: Unclear	Employed: employee: 4307 employer: 7733–8569 Self-employed: Unclear	Employed: employee: 3555 employer: 6340–6758 Self-employed: Unclear	Employed: employee: 3513 employer: 6464–6582 Self-employed: Unclear
**Covered services**	Inpatient + commonly used medicine	Outpatient + Inpatient	Inpatient	Outpatient + Inpatient	Outpatient + Inpatient	Outpatient + Inpatient		
Matching with RUM’s health needs	Mismatch	Match	Mismatch	Match	Match	Match		
**Geographic consistency**	Consistent	Consistent	Consistent	Consistent	Consistent	Consistent		
**Portability, in the view of RUMWs** [64, 70, 73, 77]	No	Quite low	No	Quite low	No	Quite low	Low	Low
**Membership selectivity** [38, 64, 71]								
Employee	Low	High	Low	High	Low	High	Moderate	Moderate
Government	Low	High	Low	High	Low	High	Moderate	Moderate

^a^ Limited-payment whole-life health insurance refers to the insurance plan that has a fixed period, in which an insurance holder pays premiums for the policy. Once the holder reaches the target years, premiums are no longer required but the policy’s benefits last the insured’s entire life. Limited-duration health insurance refers to the plan with a limited duration, paid by years or less

#### Policy gap 2: forced integration of two very different insurance plans may worsen the exclusion of RUMWs

The MWHI is specially designed for migrant workers. However, it has exacerbated the fragmentation of Chinese insurance system. In the trend of integration, some regions have begun to discard the migrant insurance, and integrate it into UEBMI or merge it with other health insurances. Table 2 compares before and after the reform of MWHI in Beijing [97–99], shanghai [92–96], and Shenzhen [100–102]. MWHI is similar with NCMS in the view of insurance type, hence the difference between MWHI and UEBMI is significant, and previous comparison between NCMS and UEBMI also suit to MWHI and UEBMI. Integrated MWHI into UEBMI means a higher costs to RUMWs themselves, government and enterprises, and the selectivity motivation of government and enterprises is higher in via of UEBMI than MWHI. Therefore, before solving of the conflicts of stakeholders’ interests as well as the problems faced by RUMWs, it can be speculated that the forced integration will worsen the exclusion of RUMWs from the urban insurance system, especially for those who are treated as unskilled workers.

### Domestic and international innovative approaches to improve the effective health insurance coverage for RUMWs

#### Domestic innovation cases

Among MWHIs in China, the model used in Shanghai and Shenzhen are considered positive examples [8].

As shown in Table 2, Shanghai provided insurance coverage for RUMWs through its comprehensive insurance system before 2011. Though this model was replaced by UEBIMI in 2011, its biggest innovation was that it was based on commercial insurance. Researchers advocated that this model should be promoted as it can be well suited to RUMWs’ high mobility and low stability because commercial insurance is not restricted by region [8, 83]. However, based on our earlier review, unless the premium under this model is paid by RUMWs themselves or by the government in their flow-out place, the commercial model still does not provide a good solution to the extra-cost problem caused by RUMWs’ high mobility and low stability faced by the government and enterprises in the flow-in place.

The innovation of the Shenzhen model lies in it combination of all health insurances into one after 2014, and in meeting different people’s needs by providing optional packages. The advantages of this model are that: 1) it reduces the fragmentation between plans; 2) it overcomes the barrier of using the family as the minimum financing unit, and increases the portability of health insurance; 3) the optional packages are more compatible with the low incomes of RUMWs; 4) it covers outpatient services and meets the RUMWs’ health needs; and 5) it is financed monthly and is more compatible with RUMWs’ high mobility. However, there are some weaknesses: 1) it lacks details about the eligibility of the self-employed RUMW; and 2) similar to the Shanghai model, the extra-cost problem faced by the government and enterprises remains, caused by the RUMWs’ high mobility and low stability.

#### International experiences

A few articles compared China’s health system with those in other countries. Table 3 summarized what can be retrieved from approaches implemented in other countries or territories that face problems similar to China. In sum, in terms of migrant-workers’ problems of insurance coverage or access to health services, countries who have a national health insurance are more likely to demonstrate the advantage of their system. Establishing a separate health insurance with low premiums for migrant workers is not an approach unique to China, but other countries consider in detail the migrant workers’ characteristics, including low incomes and the need for more primary care. Based on the causes of the problems and the obstacles encountered in solving these problems, the European approach appears the most instructive for China.

**Table 3:** international approaches implementing in other countries or territory

	USA	Kerala, India	Thailand	Australia	European Union
**Objectives**	Migratory and seasonal agricultural workers (MSAW)	Migrant workers	Migrant workers	Seasonal migrant workers	Migrant workers in the EU
**Eligible conditions**	NA	Has a work-related proof	Documented or undocumented	–	NA
**What they do.**	The federal Health Resources and Services Administration (HRSA), through the Bureau of Primary Health Care (BPHC), administers approximately $5.1 billion in federal grant support to over 1400 community health centers through 10,000 clinic sites in all 50 states and territories	Awaz Health Insurance Scheme: provides health insurance and accidental death coverage for migrant workers living in the state.	1. In 2001 the Tai Ministry of Public Health set up the migrant health insurance scheme for all migrants who are not covered by social health insurance.2. A second strand of policy action on migrant health was the establishment by the public health ministry in 2003 of innovative, migrant-friendly services with the aim of improving access to health care for all migrants, whether covered by insurance or not. These included the use of volunteer community health workers, mobile clinics for migrant communities, bilingual (mostly Tai and Burmese) signposts and information in health facilities, and outreach services in the workplace	1. Medicare covers all Australian citizens, permanent residents and citizens of New Zealand for free.2. External migrant workers: Health insurance is bundled with Visa application	1. By launching the Regulation (EC) No 883/2004, and Regulation (EC) No 987/2009 of the European Parliament and of the Council, coordinates the social security systems between European members from a legal level.2. Promote the use of European Health Insurance Card (EHIC)
**Is it a separated insurance?**	–	Yes	Yes	Citizens: No; External migrant workers: NA	No
**Mandatory or voluntary**	–	Voluntary	Voluntary	–	–
**Who pays for the eligibility**	–	NA	Migrant worker, almost 455 RMB in 2015	1. Citizens: free 2. External migrant workers: self	–
**Fee for the services**	Migrant Health Centers receive funding under Section 330(g) of the Public Health Service Act and provides services regardless of their ability to pay. Individuals without health insurance will be able to pay for services based on a sliding-fee scale, and payment is based on income and household size.	Free with Awaz insurance card	CD	1. Citizens: free 2. External migrant workers: NA	–
**Management agency**	National Association of Community Health Centers (NACHC) supports health centers caring for the MSAW population at both the program and policy levels. NACHC has a Committee on Agricultural Worker Health, which is composed of approximately 30 NACHC members who represent health centers that serve the MSAW population.	Kerala Government	A specific hospital where they registered	–	Primarily the European Commission
**Covered services**	Community health centers through 10,000 clinic sites provide culturally competent and comprehensive primary and preventive healthcare to migratory and seasonal farmworkers and their families. The program also emphasizes the occupational health and safety of this population.	Hospital services in government hospital or empaneled private network hospital	1. Screening for and treatment of certain communicable diseases. 2. Benefit package covers comprehensive curative services, including antiretroviral therapy, and a range of prevention and health promotion services, similar to the Tai universal health coverage scheme.	NA	Same with local residents
**Legal Basis**	Migrant and Seasonal Agricultural Worker Protection Act		NA	NA	Regulation (EC) No 883/2004, and Regulation (EC) No 987/2009 of the European Parliament and of the Council
**Results**	In 2017, health centers served 972,251 migrant and seasonal farmworkers and their families, of which, 872,565, or approximately 90%, were served by Migrant Health Centers	Migrant laborers working in hotels, footwear sector, and other industries can obtain this insurance card by enrolling in this scheme.	NA	NA	NA

Migrant workers are common in the EU [103, 104]. The biggest feature of the EU approach is that they consider the difference between countries; they steer clear of building one European system for all, but enhance the coordination among members from a legal level. The aim of regulations in the EU is to determine which national legislation applies to a migrant worker in all possible cases, and to avoid a situation where migrant workers are either insured in more than one Member State or not at all. The regulations have the following characteristics: 1) detailed explanations. For instance, article one of the regulations exhaustively enumerates and describes the definition of 27 related terms; 2) avoiding ambiguity. For instance, because of the situation, people may have business locally but not be employed locally. The definitions the regulation offer are “activity as an employed person” and “activity as a self-employed person” rather than definitions of a “worker” or “self-employed person”; 3) only providing the principles and leaving space for the member states, but the contents involved are comprehensive. For instance, contents include how to treat migrant workers, the rights and interests to be guaranteed, how to handle people who are double covered by multiple countries or people not covered by any country, how to solve the problem of reimbursement for medical treatment in different areas, how to deal with the cumulative of the set period, and how to cooperate and exchange between institutions or countries. Therefore, the European Commission not only provides guidance but more importantly offers coordination.

## Discussion

This systematic review reveals four important reasons behind the barriers to effective health insurance coverage for Chinese RUMWs. First, despite a decade of health care reforms, the Chinese health system is still greatly fragmented, which directly causes the low portability of SHI. Second, existing policies are not well compatible with RUMWs’ inherent characteristics and health needs. Third, local governments and enterprises have a strong intention to provide full employment only to those RUMWs with the skills that they need; whereas for other RUMWs, without stable and full employment, they cannot be included in the healthcare insurance schemes in urban areas. Fourth, due to the results outlined above, RUMWs often suffer from high working mobility and low job stability and thus become more and more disadvantaged and marginalized, socially and economically, all of which work together, placing them in a vulnerable position.

The question is: how to change such a devastating situation for RUMWs in China? Here we propose three strategies.

### Increase the health insurance portability by reducing fragmentation

The fragmentation of the Chinese health system has been discussed by many researchers [6, 28, 105, 106]. To further this understanding, we divide the concept of fragmentation into two parts: differentiation and coordination. The former focuses on the differences among departments, regions, or institutions; and the latter focuses on the compatibility and consonance among them. Evidently, differentiation and coordination are mutually influenced by each other.

In China, a variance among different regions is evident, through the view of economic capacity or the institutional settings. To reduce fragmentation, the central government of China has to take more responsibility for coordination. How can this be achieved? The European experience is instructive here: by giving more details of policy. For China, the details should include: 1) methods to handle the amount of the risk-sharing account of UEBMI and the amount funded by government in NCMS; 2) methods to resolve the conflicts of interest between regions; and 3) addressing the issue of self-employed RUMWs.

In fact, the central government has already played the role of a coordinator, as the three main SHIs were managed by one agency. China’s experience as a coordinator is evident in the raising of risk-sharing of NCMS from the county/municipal level to provincial level, as well as the implementation of reimbursements beyond jurisdictions. These examples indicate that the Chinese government has the potential to act as a good coordinator [104]. However, this has only happened within provinces, or between provinces, dependent on their willingness. A unified coordinator from the central government, as in the European Commission, is still absent.

Adopting the role of coordinator formally would increase the coordination between regions, and simultaneously weaken the influence of differentiation. To decrease the differentiation through integration, it is better to include the RUMWs into URBMI or NCMS in the flow-in area rather that integrate the MWHI into UEBMI.

### Make the policy more compatible with the characteristics and health needs of RUMWs

Several contradictions or key problems need to be solved in the future: 1) *if the RUMWs are included into the SHI in the flow-in area*, the mismatch between the financing unit of NCMS or URBMI and the migrant unit of RUMW, as well as the mismatch between the yearly finance period and RUMW’s high place mobility. Shenzhen’s model is instructive in this approach; (2) *when including the RUMWs into MWHI*, ensuring that they are covered by outpatient services and that the funds contributed by RUMWs are transferable after they go back to their hometowns; 3) *if keeping RUMWs covered by the NCMS in their flow-out area,* ensuring that NCMS covers primary care out of the jurisdictions and not only inpatient care.

### Strengthen supervision on employers and create more opportunities for RUMWs

Regarding the unwillingness of the local governments and business sectors to provide health insurance to all RUMWs, more detailed directions from the central government should be given to the local ones; meanwhile, the government should also strengthen the supervision [64], especially on private enterprises [58, 91, 107–110] and SMEs [68]. It is equally important to increase RUMWs’ ability to negotiate with their employers by offering them more substantial or informative assistances [91], offering them more vocational training to reduce their mobility, increase their job stability [91, 111, 112] and their willingness to settle in the cities [64, 65, 71, 90].

## Conclusions

Currently, policy reforms in China are not favorable to RUMWs. The number of RUMWs almost accounts for one-fifth of China’s total population, and around 90% of them are covered by health insurance. However, in relation to insurance cover in their flow-in areas, this percentage reduced to only about 20%. In this study, we summarized why and how RUMWs was selectively included into the local health insurance. By focusing on health insurance portability and fragmentation, we summarized why and how there is a mismatch of the existing insurance with RUMWs’ characteristics or health needs, as well as the game among stakeholders that place RUMWs between the cracks, without much space to choose. By sorting out and comparing current policies, we summarized why current policy reform in China is not favorable to RUMWs. We also summarized domestic and international innovative approaches that can be helpful for increasing the effective coverage on RUMWs. A series of theoretical analysis and derivation were also conducted with the aim of improving the effective coverage of health insurance for RUMWs, and the primary suggested strategies were recommended.

A few limitations of this study could be addressed in future research. First, a lack of quantitative data impeded the provision of more detailed suggestions. For instance, we emphasized that the government should increase supervision of enterprises and job training for RUMWs, but we could not clearly point out which enterprises and what kinds of training. Second, we did not focus on information related to RUMWs’ age, gender, education, and migration between or within provinces. Studies have indicated the influence of these factors on the concept and attitude towards health insurance [30, 31, 88, 113–115], but the inconsistent results are difficult to synthesize. Third, in the search of fundamental reasons, this study was not only limited to the health sector itself, but also investigated the problem from a broader view of socioeconomic and institutional structures. However, a broader view requires further studies from cross-cutting scholars. Fourth, we found that RUMWs have worse mental health than local people, and their health needs are delayed, but we failed to provide suggestions on how to cover their mental health and how to handle their delayed health need, due to a lack of related studies.

This is still the first study which systematically summarized the barriers faced by RUMWs in being effectively included by health insurance, and simultaneously discussed how to overcome existing barriers. This study will be helpful of not only for China’s UHC business, but also other countries’, as the barriers faced by migrant workers share commonalities internationally.

## Supplementary information

**Additional file 1.** Search Strategy and study selection

## Data Availability

Not applicable.

## Abbreviations

CASP: Critical appraisal skills programme
CERQual: Confidence in the evidence from reviews of qualitative research
GDP: Gross domestic product
GRADE: Grading of recommendations assessment, development, and evaluation
MWHI: Migrant work health insurance
NCMS: New-rural cooperative medical scheme
NIH: National institutes of health
PRISMA: Preferred reporting items for systematic reviews and meta-analysis
RUMWs: Rural-to-urban migrant workers
SHI: Social health insurances
SMEs: Small and medium-sized enterprises
SQAT: Study quality assessment tools
UEBMI: Urban employee-based basic medical insurance
UHC: Universal health coverage
URBMI: Urban resident-based basic medical insurance
WHO: World Health Organization

## Footnotes

1 Development of search strategy and selection criteria, study selection, quality assessment, as well as data extraction were conducted independently by two authors (SC, QY), with discussions with a third author (LX) until a consensus was reached in the case of discrepancies. The detailed process can be found in supplement s1.

2 The hukou system in china was established in the 1950s. It split people into different type (agricultural vs. non-agricultural) and location (rural vs. urban in different administrative areas), and laid the foundation of institutional pillar.
