# Supplementary material for: Barriers of effective health insurance coverage for rural-to-urban migrant workers in China: a systematic review and policy gap analysis
Source: BMC Public Health. 2020 Mar 30;20:408. doi: 10.1186/s12889-020-8448-8 (PMC7106835; doi:10.1186/s12889-020-8448-8)
Supplement: Supplementary file 1 — Additional file 1. Search Strategy and study selection [file 12889_2020_8448_MOESM1_ESM.docx]

**Supplement s1**

# Search Strategy and study selection

## Search Strategy

For the systematic review, we used the steps recommended in the Preferred Reporting Items for Systematic Reviews and Meta-Analysis (PRISMA) guidelines. We searched PubMed, Embase, Medline, Web of Science, PsycINFO, Maternity and Infant Care Database MIDIRS, the Cochrane Library, WHO Library Database (WHOLIS), WHO Global Health Library, World Bank eLibrary, OpenGrey, Zhiwang (知网, a Chinese database), and Wanfang (万方, a Chinese database) for published or unpublished papers and reports in English or Chinese between Jan 1, 2008 and Dec 31, 2018. We chose to search for studies published after Jan 1, 2008 because the situation in China has been changing rapidly, and the information provided by older studies may already have lost their background and be less useful for further work. The search terms used controlled vocabulary and free text, and included combinations intended to capture the Nongmingong (农民工, in Chinese) and rural-to-urban migrants (in English, eg, Migra* or Transient* or Emigra* Peasant* or Newcom* or New-com* or "Mobil* population" or "Mobil* people" or "Mobil* work*" or "Float* population*" or "Float* people" or "Float* work*). A detailed research strategy was developed based on previous studies^1-4^.

## Study Selection

The UHC is an all-encompassing concept. However, in this review we just focused on health insurance related issues on rural-to-urban migrants.

**The inclusion criteria are:**

1. In view of rural-to-urban migrants, offering the following information^5-7^:
   1. Accessibility: the relationship between the location of health service providers and the location of patients, taking account of patients’ transportation resources, including travel time, travel distance, and travel cost^8^. The relationship between the manner in which the health resources are organized to accept patients (including appointment systems, hours of operation, and telephone services) as well as the patients' ability to accommodate to these factors and the patients' perception of their appropriateness.
   2. Affordability: the relationship of prices of health services and providers' insurance or deposit requirements to the patients' income, ability to pay, and existing health insurance^8-10^. Patients’ perception of worth relative to total cost is a concern here, as its patients' knowledge of prices, total cost and possible credit arrangements.
   3. Acceptability: Referring to respect for the culture of individuals, minorities, peoples and communities, sensitive to gender and life-cycle requirements, as well as being designed to respect confidentiality and improve the health status of those concerned. Referring to specific patients’ reaction to such health service provider attributes such as age, sex, ethnicity, type of facility, or religious affiliation of facility or provider^11^. In turn, health service providers may have attitudes about the preferred attributes of patients or their financing mechanisms. Providers either may be unwilling to serve certain types of patients (e.g., welfare patients), or through accommodation, make themselves more or less available.
   4. Availability: the relationship of the volume and type of existing services (and resources) to the patients' volume and types of need. It refers to the adequacy of the supply of physicians, dentists, and other providers; of facilities such as clinics and hospitals; and of specialized programs and services such as mental health and emergency care^8,10,11^.
2. In the view of health system, offering the following information (Six blocks theory^12^):
   1. Health service delivery: it refers eight key aspects or characteristics, including comprehensiveness, accessibility, coverage, continuity, quality, person-centeredness, coordination, and accountability & efficiency^12^.
   2. Health workforce: it can be defined as “all people engaged in actions whose primary intent is to enhance health”^3^. These human resources include clinical staﬀ, such as physicians, nurses, pharmacists, and dentists, as well as management and support staﬀ, i.e. those who do not deliver services directly but are essential to the performance of health systems, such as managers, ambulance drivers, and accountants^12^.
   3. Health information system: it is sometimes equated with monitoring and evaluation. In addition, the information system also serves broader objectives, such as providing alerts and early warning capability, supporting patient and health facility management, enabling planning, underpinning and stimulating research, permitting health situation and trends analyses, orienting global reporting, and reinforcing communication of health challenges to diverse users^12^.
   4. Access to essential medicines: essential medicines are intended to be available within the context of functioning health systems at all times, in adequate amounts, in the appropriate dosage, with assured quality, and at a price that individuals and the community can aﬀord .
   5. Financing: refers to the function of a health system concerned with the mobilization, accumulation, and allocation of money to cover the health needs of the people, individually and collectively, in the health system^12^.
   6. Leadership/government: it involves ensuring that strategic policy frameworks exist and are combined with eﬀective oversight, coalition-building, regulation, attention to system design, and accountability. Accountability here is an intrinsic aspect of governance that concerns the management of relationships between various stakeholders in health, including individuals, households, communities, firms, governments, nongovernmental organizations, private firms, and other entities that have the responsibility to finance, monitor, deliver, and use health services. Accountability involves: delegation or an understanding (either implicit or explicit) of how services are supplied; financing to ensure that adequate resources are available to deliver essential services; performance around the actual supply of services; receipt of relevant information to evaluate or monitor performance; enforcement, such as imposition of sanctions or the provision of rewards for performance^12^.

**The excluded criteria are:**

1. Not related to health
2. Not focused on mainland China
3. Only used the quantitative or qualitative data collected before 2008
4. Only focus on the left-behind children, or migrant’s children in flow-in region
5. Only focus on the left-behind elderly
6. Only focus on maternity Insurance
7. Only focus on employment injury insurance
8. Only focus on occupational risk
9. Only focus on living conditions
10. Only focus on using of condom
11. The measure of prevalence and incidence of diseases
12. Not an original study, including review and meta-analysis.
13. Has neither quantitative data nor qualitative data, e.g. theory analysis.
14. Comments, correspondence, editorials, and perspective pieces
15. Introduction of a program, a policy, or a conference
16. No full text available, e.g. conference abstract
17. Clinical trial, including intervention evaluation and medicine evaluation
18. Development of evaluation indicators
19. Development of the scale tool
20. Focus on the migrants, but can’t separate the results of rural-to-urban migrants
21. Quality of Quantitative study was ranked as “quite low”

In the first-round, each title and abstract was screened by two independent reviewers (SC, QY), all relevant, possibly relevant, and cannot be determined studies were included. In the second-round, each available full text was reviewed by the two independent reviewers using standardized inclusion criteria (details are available in next part of quality assessment). Quantitative studies whose quality were ranked as “quite low” were excluded. All relevant qualitative studies were included even if their quality were ranked as “quite low”, as the number of available qualitative studies were few. Discrepancies in the inclusion or exclusion of papers during screening were discussed with a third reviewer (LX) until consensus was achieved.

## Quality Assessment of study

The quality of the observational cohort and cross-sectional studies was assessed using an adaptation of the Study Quality Assessment Tools (SQAT) developed by the US National Institutes of Health (NIH)^13^. The assessment included the following domains: research question, study population, eligibility criteria, participation rate, sample size justification, exposure assessed prior to outcome measurement, sufficient timeframe, different exposure level, exposure measure & assessment, repeated exposure assessment, outcome measures, blinding of outcome assessors, follow-up rate, and statistical analysis.

The quality of the case-control studies was also assessed using SQAT^13^, and the assessment included the following domains: research question, study population, target population and case representation, sample size justification, uniform eligibility criteria, case and control definitions, random selection, concurrent controls, exposure assessed prior to outcome measurement, exposure measure and assessment, blinding of outcome assessors, and statistical analysis.

The quality of the qualitative studies was assessed using an adaptation of the Critical Appraisal Skills Programme (CASP) quality-assessment tool^14^, and the assessment included the following domains: aims, methodology, research design, recruitment strategy, data collection, reflexivity, ethical considerations, data analysis, findings statement, and research contribution.

The quality of the mixed-methods studies was assessed twice separately using the above tools.

These adopted tools give three-level assessment for the overall quality. To better distinguish the studies, especially the quantitative studies, in this study a four-level assessment of overall quality, “high,” “moderate”, “low”, or “quite low”, was rated. The overall quality assessment was based on independent evaluation by two reviewers (SC, QY), and discussion with a third reviewer (LX) until consensus was reached in the case of discrepancies.

## Quality Assessment of finding

The overall quality assessment of findings was based on independent evaluation by two reviewers (SC, QY), and discussion with a third reviewer (LX) until consensus was reached in the case of discrepancies.

### Quantitative finding

Each quantitative finding was assessed using the GRADE (Grading of Recommendations Assessment, Development, and Evaluation) approach. GRADE offers a transparent and structured process for developing and presenting evidence summaries for systematic reviews and guidelines in health care and for carrying out the steps involved in developing recommendations^15^. This approach is designed for reviews and guidelines that examine alternative management strategies or interventions, which may include no intervention or current best management. The GRADE approach includes five elements:

- Risk of bias. What are the study limitations? Risk of bias was assessed using the NIH-SQAT.
- Inconsistency. It usually refers to heterogeneity, which means any kind of variation across studies.
- Indirectness. It refers to how well the evidence included in the review answers the review question, including indirect population, intervention, comparator, outcome, or comparison.
- Publication bias: Are these all of the relevant studies? The risk of publication bias is often higher for reviews that include only small trials, as larger trials are less likely to be unpublished or unknown, irrespective of their actual findings.
- Imprecision. How precise is the effect size? Results are imprecise when studies include only relatively few patients or for dichotomous outcomes, there are few events, or when there is a lot of variation in the effects among the participants in continuous measures. As a result, there may be wide confidence intervals (CIs) around the effect estimate.

We assessed each of these five components as being “not serious”, “serious”, or “very serious” regarding the specific component. In the original GRADE approach, randomized controlled trials (RCTs) start as high-quality evidence and observational studies as low-quality evidence. The above five factors are used to rate down the quality of evidence and another three factors are used to rate up. The three rating up factors are large magnitude of effect, dose response, and confounders likely minimize the effect. Ultimately, the quality of evidence for each finding falls into four categories: “high”, “moderate”, “low”, or “quite low”.

As there are no RCT studies in our topic of interest, this original rating approach will lead the quality of evidence mostly falls into “low” or “quite low”. To better distinguish the quantitative findings, in this study we start the observational studies with moderate-quality evidence.

### Qualitative finding

Each qualitative finding was assessed using the GRADE-CERQual (Confidence in the Evidence from Reviews of Qualitative Research) approach. GRADE-CERQual is a method to transparently assess and describe how much confidence to place in findings from systematic reviews of qualitative evidence^16^. The confidence here is an assessment of the extent to which a review finding is a reasonable representation of the phenomenon of interest, for instance the phenomenon of interest is unlikely to be substantially different from the review finding. The GRADE-CERQual approach includes four elements:

- Methodological limitations. The extent to which there are concerns about the design or conduct of the primary studies that contributed evidence to an individual review finding. Methodological limitations were assessed using the CASP tool.
- Coherence. An assessment of how clear and cogent the fit is between the data from the primary studies and a review finding that synthesises that data.
- Adequacy of data. An overall determination of the degree of richness and quantity of data supporting a review finding.
- Relevance. The extent to which the body of evidence from the primary studies supporting a review finding is applicable to the context (perspective or population, phenomenon of interest, setting) specified in the review question.

We assessed each of these four components as being “quite low”, “low”, “moderate”, or “high” regarding the specific component. Based on an overall assessment of these four components, the confidence in the evidence for each review finding was assessed as “quite low”, “low”, “moderate”, or “high”.

## Data Extraction

Data was extracted using a standardized form including the following domains: study setting, sample characteristics, objectives, design, data collection and analysis methods, and conclusions. Themes, findings, and participant quotations were extracted from qualitative studies. Data source, outcome measures, and results were extracted from quantitative studies.

Two reviewers (SC, QY) extracted all study data. A third reviewer (LX) arbitrated any discrepancies between the first two reviewers.

## PubMed

(((((((((((((((((((((("Human Migration"[MeSH Terms]) OR ("Transients and Migrants"[MeSH Terms])) OR ("Emigration and Immigration"[MeSH Terms])) OR Migra*[Title/Abstract]) OR Transient*[Title/Abstract]) OR Emigra*[Title/Abstract]) OR Immigra*[Title/Abstract]) OR Foreign*[Title/Abstract]) OR Peasant*[Title/Abstract]) OR Refug*[Title/Abstract]) OR Newcom*[Title/Abstract]) OR new-com*[Title/Abstract]) OR "Mobil* population"[Title/Abstract]) OR "Mobil* people"[Title/Abstract]) OR "Mobil* work*"[Title/Abstract]) OR "Float* population*"[Title/Abstract]) OR "Float* people"[Title/Abstract]) OR "Float* work*"[Title/Abstract])) AND ((China[Title/Abstract]) OR Chinese[Title/Abstract])) AND ("2008/01/01"[Date - Publication] : "2018/12/31"[Date - Publication])) AND ((English[Language]) OR Chinese[Language])) NOT Review[Publication Type]

**Hits: 7639**

## Embase

|  | **Search strategy** | **Hits** |
| --- | --- | --- |
| 1 | (Migra* or Transient* or Emigra* or Foreign* or Peasant* or Refug* or Newcom* or New-com* or "Mobil* population" or "Mobil* people" or "Mobil* work*" or "Float* population*" or "Float* people" or "Float* work*" or Immigra*).af. | 1,096,119 |
| 2 | limit 1 to yr="2008 - 2018" | 612,429 |
| 3 | China.ti. or China.ab. or China.kw. | 172,910 |
| 4 | Chinese.ti. or Chinese.ab. or Chinese.kw. | 240,466 |
| 5 | 3 or 4 | 368,046 |
| 6 | (English or Chinese).lg. not Review.pt. | 25,488,997 |
| 7 | 1 and 2 and 5 and 6 | 11,470 |

## Medline

|  | **Search strategy** | **Hits** |
| --- | --- | --- |
| 1 | (Migra* or Transient* or Emigra* or Immigra* or Foreign* or Peasant* or Refug* or Newcom* or New-com* or "Mobil* population" or "Mobil* people" or "Mobil* work*" or "Float* population*" or "Float* people" or "Float* work*").af. | 872,986 |
| 2 | limit 1 to yr="2008 - 2018" | 394,347 |
| 3 | (English or Chinese).lg. not Review.pt. | 22,851,106 |
| 4 | China.ab. or China.ti. or China.kw. or China.kf. | 144,735 |
| 5 | Chinese.ab. or Chinese.ti. or Chinese.kw. or Chinese.kf. | 196,129 |
| 6 | 4 or 5 | 303,480 |
| 7 | 1 and 2 and 3 and 6 | 8,705 |

## Web of Science

|  | **Search strategy** | **Hits** |
| --- | --- | --- |
| 1 | (TS = (Migra* OR Transient* OR Emigra* OR Immigra* OR Foreign* OR Peasant* OR Refug* OR Newcom* OR New-com* OR "Mobil* population" OR "Mobil* people" OR "Mobil* work*" OR "Float* population*" OR "Float* people" OR "Float* work*") AND TS = (China OR Chinese) NOT TI = Review) AND LANGUAGE: (Chinese) Indexes=SCI-EXPANDED, SSCI, A&HCI, CPCI-S, CPCI-SSH, BKCI-S, BKCI-SSH, ESCI Timespan=2008-2018 | 730 |
| 2 | (TS = (Migra* OR Transient* OR Emigra* OR Immigra* OR Foreign* OR Peasant* OR Refug* OR Newcom* OR New-com* OR "Mobil* population" OR "Mobil* people" OR "Mobil* work*" OR "Float* population*" OR "Float* people" OR "Float* work*") AND TS = (China OR Chinese) NOT TI = Review) AND LANGUAGE: (English) Indexes=SCI-EXPANDED, SSCI, A&HCI, CPCI-S, CPCI-SSH, BKCI-S, BKCI-SSH, ESCI Timespan=2008-2018 | 36,225 |
| 3 | 1 OR 2 | 36,955 |

## PsycINFO

|  | **Search strategy** | **Hits** |
| --- | --- | --- |
| 1 | TX Migra* OR TX Transient* OR TX Emigra* OR TX Immigra* OR TX Foreign* OR TX Peasant* OR TX Refug* OR TX Newcom* OR TX New-com* OR TX "Mobil* population" OR TX "Mobil* people" OR TX "Mobil* work*" OR TX "Float* population*" OR TX "Float* people" OR TX "Float* work*" | 150,133 |
| 2 | TI Chinese OR AB Chinese OR KW Chinese OR TI Chinese OR AB Chinese OR KW Chinese | 43,676 |
| 3 | LA English OR LA Chinese | 4,315,196 |
| 4 | 1 AND 2 AND 3  Limiters - Publication Year: 2008-2018 | 4,372 |

## Maternity and Infant Care Database MIDIRS

|  | **Search strategy** | **Hits** |
| --- | --- | --- |
| 1 | (Migra* or Transient* or Emigra* or Immigra* or Foreign* or Peasant* or Refug* or Newcom* or New-com* or "Mobil* population" or "Mobil* people" or "Mobil* work*" or "Float* population*" or "Float* people" or "Float* work*").af. | 4,367 |
| 2 | limit 1 to yr="2008 - 2018" | 2,344 |
| 3 | (China or Chinese).af. | 3,008 |
| 4 | 1 and 2 and 3 | 90 |

## The Cochrane Library

|  | **Search strategy** | **Hits** |
| --- | --- | --- |
| 1 | (Migra*):ti,ab,kw OR (Transient*):ti,ab,kw OR (Emigra*):ti,ab,kw OR (Immigra*):ti,ab,kw OR (Foreign*):ti,ab,kw (Word variations have been searched) | 24,419 |
| 2 | (Peasant*):ti,ab,kw OR (Refug*):ti,ab,kw OR (Newcom*):ti,ab,kw OR (New-com*):ti,ab,kw (Word variations have been searched) | 295 |
| 3 | ("Mobil* population"):ti,ab,kw OR ("Mobil* people"):ti,ab,kw OR ("Mobil* work*"):ti,ab,kw (Word variations have been searched) | 34 |
| 4 | ("Float* population*"):ti,ab,kw OR ("Float* people"):ti,ab,kw OR ("Float* work*"):ti,ab,kw (Word variations have been searched) | 0 |
| 5 | 1 OR 2 OR 3 OR 4 | 24,700 |
| 6 | (China):ti,ab,kw OR (Chinese):ti,ab,kw (Word variations have been searched) | 26,278 |
| 7 | 5 AND 6 with Cochrane Library publication date Between Jan 2008 and Dec 2018 | 472 |

## WHO Library Database (WHOLIS)

kw,wrdl: Migra* OR Transient* OR Emigra* OR Immigra* OR Foreign* OR Peasant* OR Refug* OR Newcom* OR New-com* OR "Mobil* population" OR "Mobil* people" OR "Mobil* work*" OR "Float* population*" OR "Float* people" OR "Float* work*" or ti,wrdl: Migra* OR Transient* OR Emigra* OR Immigra* OR Foreign* OR Peasant* OR Refug* OR Newcom* OR New-com* OR "Mobil* population" OR "Mobil* people" OR "Mobil* work*" OR "Float* population*" OR "Float* people" OR "Float* work*"' with limit(s): 'yr,st-numeric,ge=2008 and yr,st-numeric,le=2018'

**Hits:164**

## WHO Global Health Library

(tw:(Migra* OR Transient* OR Emigra* OR Immigra* OR Foreign* OR Peasant* OR Refug* OR Newcom* OR New-com* OR "Mobil* population" OR "Mobil* people" OR "Mobil* work*" OR "Float* population*" OR "Float* people" OR "Float* work*")) AND (tw:(China OR Chinese)) AND (year_cluster:(2008-2018))

**Hits: 60**

## World Bank eLibrary

|  | **Search strategy** | **Hits** |
| --- | --- | --- |
| 1 | [[All: migra*] OR [All: transient*] OR [All: emigra*] OR [All: immigra*] OR [All: foreign*] OR [All: peasant*] OR [All: refug*] OR [All: newcom*] OR [All: new-com*] OR [All: "mobil* population"] OR [All: "mobil* people"] OR [All: "mobil* work*"] OR [All: "float* population*"] OR [All: "float* people"] OR [All: "Float* work*"]] AND [[Publication Title: china] OR [Publication Title: chinese]] with publication date Between Jan 2008 and Dec 2018 | 267 |
| 2 | [[All: migra*] OR [All: transient*] OR [All: emigra*] OR [All: immigra*] OR [All: foreign*] OR [All: peasant*] OR [All: refug*] OR [All: newcom*] OR [All: new-com*] OR [All: "mobil* population"] OR [All: "mobil* people"] OR [All: "mobil* work*"] OR [All: "float* population*"] OR [All: "float* people"] OR [All: "Float* work*"]] AND [[Keywords: china] OR [Keywords: chinese]] with publication date Between Jan 2008 and Dec 2018 | 44 |
| 3 | [[All: migra*] OR [All: transient*] OR [All: emigra*] OR [All: immigra*] OR [All: foreign*] OR [All: peasant*] OR [All: refug*] OR [All: newcom*] OR [All: new-com*] OR [All: "mobil* population"] OR [All: "mobil* people"] OR [All: "mobil* work*"] OR [All: "float* population*"] OR [All: "float* people"] OR [All: "Float* work*"]] AND [[Abstract: china] OR [Abstract: chinese]] with publication date Between Jan 2008 and Dec 2018 | 600 |
| 4 | 1 OR 2 OR 3 | 607 |

## OpenGrey

(Migra* OR Transient* OR Emigra* OR Immigra* OR Foreign* OR Peasant* OR Refug* OR Newcom* OR New-com* OR "Mobil* population" OR "Mobil* people" OR "Mobil* work*" OR "Float* population*" OR "Float* people" OR "Float* work*") AND (China OR Chinese) AND (lang: "en" OR "zh") AND (year: 2008 OR 2009 OR 2010 OR 2011 OR 2012 OR 2013 OR 2014 OR 2015 OR 2016 OR 2017 OR 2018)

**Hits: 153**

## Zhiwang (知网)

SU = “农民工” OR KY = “农民工” OR TI = “农民工”

Limitation time: 2008-01-01 to 2018-12-31

Limitation database:

- Academic journal: 期刊,特色期刊,学术辑刊
- Conference journal: 国内会议
- Master dissertation: 硕士
- Doctorate dissertation: 博士

Limitation research area:

- Medicine and health: 医药卫生科技
- Philosophy and humanities: 哲学与人文科学-论理学，心理学
- Sociology: 社会科学II辑-社会学及统计学，人口学与计划生育
- Economics and management：经济与管理科学-保险，管理学，领导学与决策学，科学研究管理

**Hits: 7,026**

## Wanfang (万方)

SU = “农民工” OR KY = “农民工” OR TI = “农民工”

Limitation time: 2008-01-01 to 2018-12-31

Limitation database:

- Academic journal: 期刊论文
- Conference journal: 会议论文
- academic dissertation: 学位论文

Limitation research area:

- Medicine and health: 医药卫生
- Philosophy and humanities: 哲学宗教-心理，论理
- Sociology: 社会科学总论
- Economics and management：经济-财政、金融-保险
- Environmental science: 环境科学-安全科学

**Hits: 8,194**

# PRISMA 2009 Flow Diagram


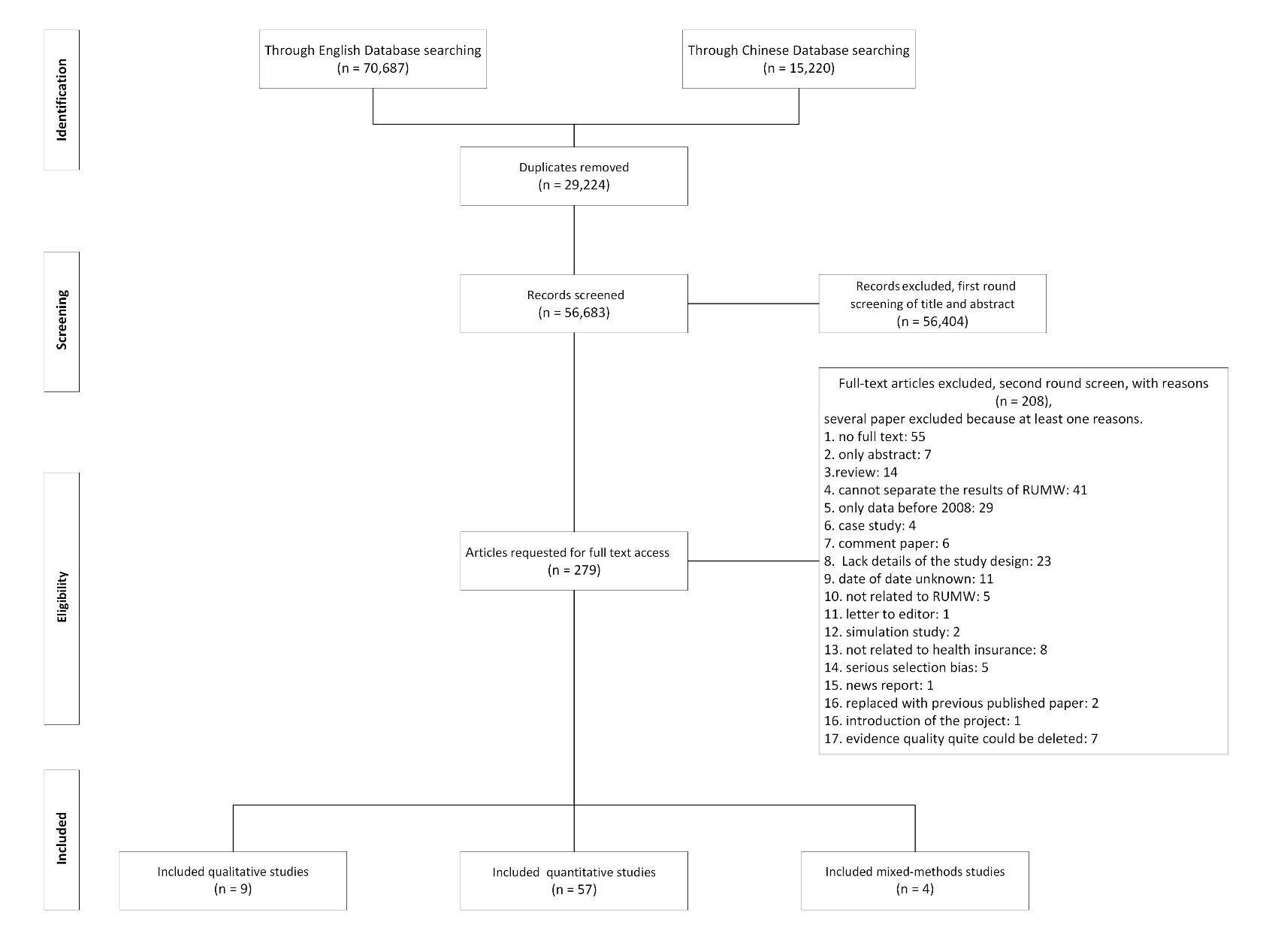


**References**

1. Schilgen B, Nienhaus A, Handtke O, Schulz H, Mosko M. Health situation of migrant and minority nurses: A systematic review. PLoS One 2017; 12(6): e0179183.

2. Seedat F, Hargreaves S, Nellums LB, Ouyang J, Brown M, Friedland JS. How effective are approaches to migrant screening for infectious diseases in Europe? A systematic review. The Lancet infectious diseases 2018.

3. Fitzgerald S, Chen X, Qu H, Sheff MG. Occupational injury among migrant workers in China: a systematic review. Injury Prevention 2013; 19(5): 348-54.

4. Markkula N, Cabieses B, Lehti V, Uphoff E, Astorga S, Stutzin F. Use of health services among international migrant children–a systematic review. Globalization and health 2018; 14(1): 52.

5. Edward J, Hines-Martin V. Examining perceived barriers to healthcare access for Hispanics in a southern urban community. Journal of Hospital Administration 2016; 5(2): p102.

6. Levesque J-F, Harris MF, Russell G. Patient-centred access to health care: conceptualising access at the interface of health systems and populations. International journal for equity in health 2013; 12(1): 18.

7. Anyinam C. Availability, accessibility, acceptability, and adaptibility: Four attributes of African ethno-medicine. Social science medicine 1987; 25(7): 803-11.

8. Peters DH, Garg A, Bloom G, Walker DG, Brieger WR, Hafizur Rahman M. Poverty and access to health care in developing countries. Annals of the New York Academy of Sciences 2008; 1136(1): 161-71.

9. Bice TW, Eichhorn RL, Fox PD. Socioeconomic status and use of physician services: a reconsideration. Medical Care 1972: 261-71.

10. Fein R. On achieving access and equity in health care. The Milbank Memorial Fund Quarterly 1972; 50(4): 157-90.

11. Donabedian A. Aspects of medical care administration: specifying requirements for health care: Harvard University Press; 1973.

12. Organization WH. Monitoring the building blocks of health systems: a handbook of indicators and their measurement strategies: World Health Organization; 2010.

13. Health UNIo. Study Quality Assessment Tools. https://www.nhlbi.nih.gov/health-topics/study-quality-assessment-tools.

14. Singh J. Critical appraisal skills programme. Journal of pharmacology Pharmacotherapeutics 2013; 4(1): 76.

15. Guyatt G, Oxman AD, Akl EA, et al. GRADE guidelines: 1. Introduction—GRADE evidence profiles and summary of findings tables. Journal of clinical epidemiology 2011; 64(4): 383-94.

16. Lewin S, Booth A, Glenton C, et al. Applying GRADE-CERQual to qualitative evidence synthesis findings: introduction to the series. BioMed Central; 2018.
